# Ultrasensitive detection of endocrine disruptors via superfine plasmonic spectral combs

**DOI:** 10.1038/s41377-021-00618-2

**Published:** 2021-09-07

**Authors:** Lanhua Liu, Xuejun Zhang, Qian Zhu, Kaiwei Li, Yun Lu, Xiaohong Zhou, Tuan Guo

**Affiliations:** 1 State Key Joint Laboratory of ESPC, School of Environment, Tsinghua University, Beijing, 100084 China; 2 Institute of Photonics Technology, Jinan University, Guangzhou, 510632 China

**Keywords:** Imaging and sensing, Biophotonics, Fibre optics and optical communications

## Abstract

The apparent increase in hormone-induced cancers and disorders of the reproductive tract has led to a growing demand for new technologies capable of detecting endocrine disruptors. However, a long-lasting challenge unaddressed is how to achieve ultrahigh sensitive, continuous, and in situ measurement with a portable device for in-field and remote environmental monitoring. Here we demonstrate a simple-to-implement plasmonic optical fiber biosensing platform to achieve an improved light–matter interaction and advanced surface chemistry for ultrasensitive detection of endocrine disruptors. Our platform is based on a gold-coated highly tilted fiber Bragg grating that excites high-density narrow cladding mode spectral combs that overlap with the broad absorption of the surface plasmon for high accuracy interrogation, hence enabling the ultrasensitive monitoring of refractive index changes at the fiber surface. Through the use of estrogen receptors as the model, we design an estradiol–streptavidin conjugate with the assistance of molecular dynamics, converting the specific recognition of environmental estrogens (EEs) by estrogen receptor into surface-based affinity bioassay for protein. The ultrasensitive platform with conjugate-induced amplification biosensing approach enables the subsequent detection for EEs down to 1.5 × 10^−3^ ng ml^−1^ estradiol equivalent concentration level, which is one order lower than the defined maximal E_2_ level in drinking water set by the Japanese government. The capability to detect EEs down to nanogram per liter level is the lowest limit of detection for any estrogen receptor-based detection reported thus far. Its compact size, flexible shape, and remote operation capability open the way for detecting other endocrine disruptors with ultrahigh sensitivity and in various hard-to-reach spaces, thereby having the potential to revolutionize environment and health monitoring.

## Abstract (web summary)

An optical fiber biosensor displaying superfine plasmonic spectral combs and enhanced by conjugate-induced amplification enables the detection of environmental estrogens down to pg/mL estradiol equivalent concentration level.

## Graphical abstract

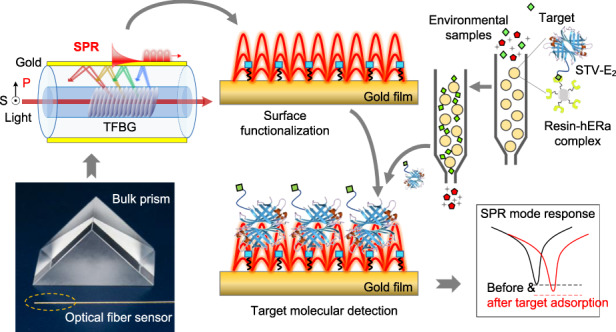

## Introduction

Environmental estrogens (EEs), as typical endocrine disruptors, are structurally diverse compounds that can interact with nuclear estrogen receptors (nERs) and pose significant risks to human and ecological health^1^. A large number EEs are improperly released into the environment due to human activities (e.g., industrial, agricultural, urban), especially the huge estrogenic drug consumption world widely^2,3^. As a consequence, EE contamination has been listed as one of the global environmental issues to be addressed through international collaboration by the United Nations. A variety of technologies have been applied in the field of estrogen detection (Fig. S1). Because of the structural diversity of EEs, it is difficult to detect all broad ranges of EEs by using many conventional structure-specific instrumental analytical methods, such as high-performance liquid chromatography–mass spectrometry and gas chromatography–mass spectrometry. Notably, these methods are powerless to the unknown ones. Therefore, developing the advanced and powerful detection technologies to characterize as many EEs as possible in the environment is still challenging, however, highly demanded.

By mimicking the function of the natural estrogens or disturbing the signal transduction pathways for estrogens, EEs could induce abnormality in the growth, development, and maintenance of the reproductive system^4,5^. nERs are members of the steroid receptor family of nuclear receptors and mediate most estrogen responses in the body. The binding interaction between EEs and nERs is identified as one of the molecular initiative events of estrogen signal transduction pathways, leading to the subsequent downstream events, such as nER-mediated cell proliferation^6–8^. Based on the specific interactions between EEs and nERs, many cell-based in vitro assays are established, e.g., ER-CALUX®, the MELN, and the Yeast Estrogen Screen (YES) assays, to provide effective and robust detection of broad ranges of EEs^9^. However, the majority of these bioassays require days-long operations, specialized experts, or sophisticated equipment. Notably, the cell-based bioassays are confronted with the problem of cytotoxic substances, which may be present in environmental samples and could lead to erroneous results unless proper controls are included^10^.

As an alternative to cell-based bioassays, cell-free approaches offer in vitro protein expression for analytic purposes. By using nER protein as a recognition element, the competitive binding assays have also been established by using radioactive estradiol (17β-Estradiol (E_2_)), such as [3H]E_2_^11,12^ or [125I]E_2_^13^, as the signal reporter. It is worth mentioning that such competitive nER binding in vitro experiment has been listed as the first scheme of endocrine-disruptor screening program by the US Environmental Protection Agency. However, the reported nER competitive binding assays mostly rely on the homogeneous system, and the real-time, continuous, and intelligent detection of EEs has not yet been fully developed. With the flourishing development of materials science and synthetic biology, biosensor technology is expected to break through the above issues.

To date, numerous nER-based biosensors have been developed by using different signal transductions modes, such as fluorescence^14^, surface plasmon resonance (SPR)^15–17^, piezoelectric^18^, and electrochemistry^19,20^. These cell-free biosensors are cost-effective and robust to the cytotoxicity of environmental samples. They are useful complements to existing laboratory methods, facilitating quick and affordable identification of all EEs that act through the nERs in remote, non-laboratory settings^1,14^. However, most of them are still blamed with inferior sensitivities largely due to the binding affinity limitation of nER protein, especially used for the analysis of EEs commonly existing at trace concentrations in the environment. As an example, the defined maximal E_2_ level in drinking water is set to be 0.08 ng ml^−1^ by the Japanese government^21^. Hence, developing an advanced and sensitive nER-based biosensing platform is highly demanded, however, still challenging.

Recently, a new promising class of label-free optical biosensors, i.e., fiber-optic SPR biosensor, has emerged due to their desirable features, such as the suitability to miniaturization, remote sensing, and ease of use compared with the well-established bulky prism configurations (i.e., Kretschmann–Raether prism)^22–24^. These features enable it possible to be inserted into various hard-to-reach environments for in situ detection either as a hand-held probe or as a set of remotely operated devices fixed at various locations along a fiber-optic cable. The most common fiber-optic SPR sensors are made from cladding-modified optical fibers (unclad, side-polished, tapered, and U-shaped) covered with a nanometric layer of gold or silver^25^. More recently, the fiber grating technology based on tilted fiber Bragg grating (TFBG) has been developed for the construction of a highly efficient SPR sensor with several unique advantages^26,27^. Such sensor provides an additional resonant mechanism of high-density narrow cladding mode spectral combs at the near-infrared wavelength (with a spectral width of the resonance between 0.01 and 0.1 nm) that overlaps with the broad absorption of the surface plasmon for high accuracy interrogation, which rises the resolution of refractive index (RI) from 10^−6^ to 10^−8^ RIU (refractive index unit) and offers a linear RI response in both air and liquids^28,29^. Second, the mechanical resistance of the TFBG sensor is minimally impacted and its mass production with good reproducibility can be easily achieved by well-established phase masks for grating inscription. Last but not least, the temperature cross-sensitivity can be suppressed by calibrating the light remaining in the core of the TFBG fiber, i.e., the Bragg resonance, which is inherently insensitive to the external RI changes unless acting as a thermometer. Even with the excellent features mentioned above, the research on TFBG-based fiber-optic SPR technology used for ultrasensitive detection of broad ranges of EEs via ER has never been reported. The robust biosensor design with advanced surface chemistry of TFBG-based fiber-optic and rational biosensing strategy for EEs is still the challenge to be addressed.

To address the above research gap, we developed a gold film-coated TFBG-based SPR biosensor for the ultrasensitive screening of EEs. Through the covalent surface modification of plasmonic fiber optic, the specific interaction of nERs and EEs was converted into measurable signals to realize the facile detection of EEs in environmental samples. Benefiting from the ultrahigh sensitivity of gold film-coated TFBG-based SPR biosensing platform and the broad recognition capability of nERs, the water samples containing EEs in the nanogram per liter estradiol equivalent concentration (EEQ) range were determined by the biosensor with satisfactory recovery rates. Such sensor offers an ideal solution to meet the strategic layout of continuous assessment of global environmental endocrine disruptors.

## Results

### Characterization of plasmonic optical fiber sensor

To achieve specificity of detection, functionalization of plasmonic optical fiber to effectively discriminate the E_2_–streptavidin (STV) was indispensable. The well-established strategy to functionalize functional groups/molecules onto gold film-coated SPR surface is via Au-S bond by using thiol-end organic compounds to form self-assembled monolayers (SAMs) spontaneously^30^. Desthiobiotin–polyethylene glycol–thiol (DTB-PEG-SH) was used to form SAMs on gold surface, which offered the exposed DTB to bind the released E_2_–STV conjugate efficiently, while the bovine serum albumin (BSA) solution as a blocking agent was incubated to block the nonspecific binding sites and enhance the specific STV–DTB interaction, and hence increase the signal/noise ratio. Moreover, the PEG linker between -SH and DTB offered better water solubility and reduced the steric hindrance to enhance the reactivity of DTB.

**Fig. 1:**
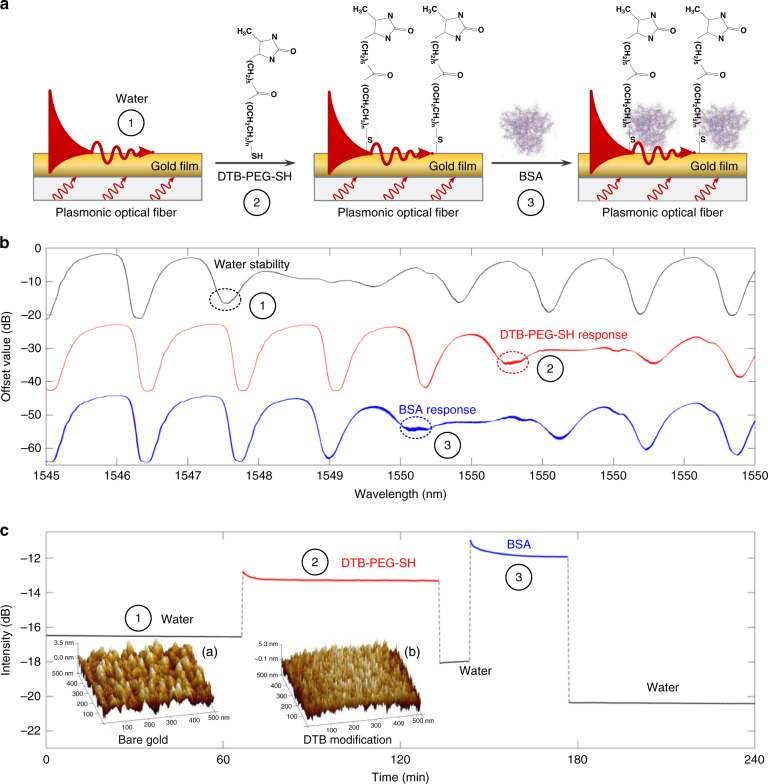
Schematic illustration and characterization of the surface chemical modification process. **a** Schematic illustration of immobilizing the DTB-PEG-SH and BSA molecules on the fiber surface; **b** reflected spectra collected every 20 s for stability in water (66 min) and for real-time monitoring in DTB-PEG-SH (67 min) and BSA (33 min) solution; **c** the response curves of gold film-coated TFBG for the marked modes ①, ②, and ③, i.e., intensity variation as a function of time during the water stability, DTB-PEG-SH, and BSA functionalization processes. Inset shows 3-D AFM topographic images of (a) bare and (b) DTB-modified plasmonic optical fiber.

The functionalization of the Au-coated TFBG fiber followed the procedures below, as shown in Fig. 1a. First, the probe was immersed in the deionized (DI) water for 66 min to stabilize this sensor, which can eliminate environmental interference to verify the stability of sensing system. Second, bare gold was functionalized with DTB-PEG-SH crosslinker and then coated with the BSA blocker for 67 and 33 min, respectively. Notably, the DI water was used to rinse the probe for 10 min to remove the residuals at the end of both procedures of DTB-PEG-SH and BSA modification. Figure 1b shows the variable spectra recorded every 20 s under the above three different conditions: pure gold film-coated TFBG in water (black curve); gold film-coated TFBG modified with DTB-PEG-SH (red curve) and then blocked with BSA solution (blue curve). Three modes with the highest sensitivity were selected, respectively, and marked as ①, ②, and ③. Based on the above results, the intensity variations of gold film-coated TFBG as a function of time during the water stability and the DTB-PEG-SH and BSA adsorption on gold surface, respectively, are demonstrated at each selected mode and compared in Fig. 1c. Its inset shows the atomic force microscopic (AFM) images of the morphology of bare and DTB-PEG-SH-modified plasmonic optical fiber. Extensive analysis of the AFM topography cross-sections (Fig. S2) showed that the surface thickness of the chemically modified fiber was slightly increased than that of the bare fiber considering the small size of the DTB-PEG-SH. The response curve versus time also clearly demonstrated that the intensity was stable at the selected highly sensitive mode ① in water while changed when the TFBG fiber was modified by DTB-PEG-SH and BSA.

### Design of E_2_–STV conjugate

The length and structure of the linker between the E_2_ and STV are critical factors affecting the binding performance of the E_2_-STV conjugate and hERα ligand-binding domain (LBD). Although the flexible and rigid linkers in the design of estradiol derivatives were considered, the ligands with rigid linkers showed unavoidable atomic position conflict with hERα LBD, indicating that the rigid structure was not suitable for the linker design for E_2_-STV conjugates (Fig. S3). Among estradiol derivatives with flexible linkers, estradiol derivative 4 was docked to hER-LBD as shown in Fig. S4, and the -COOH terminal was blocked in the hERα LBD, showing that the linker was too short to meet the requirement of connecting STV, so that no further simulation will be performed in the follow-up.

**Fig. 2:**
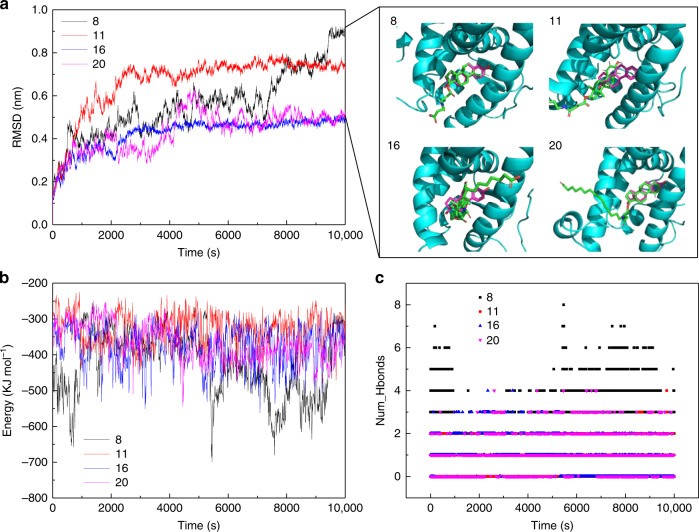
Design of E_2_–STV conjugate based on the MD simulations. **a** Time evolution of RMSD for the backbone atoms of hERα LBD and estradiol derivatives 8, 11, 16, and 20 with different flexible carbon skeleton as shown in Table 1 (left) and the snapshot of the estradiol derivative–hERα LBD complex conformation at the end of MD simulation (right). In the right figure, E_2_ in magenta is used as a position reference for conformations; **b** total non-bond energy of estradiol derivative and hERα LBD complexes; **c** Hbond number of estradiol derivatives and hERα complexes.

Figure 2 summarizes the molecular dynamics (MD) simulation results of carbonylated estradiol derivatives, named 8, 11, 16, and 20 with different numbers of flexible carbon skeleton as shown in Table 1. The root-mean-square deviation (RMSD) is an accepted index to measure the conformational changes of the MD simulation systems. Figure 2a displays the time evolution of RMSDs for hERα LBD in the simulations with four carbonylated estradiol derivatives, respectively. As expected, a plateau was reached with the RMSDs of all complexes <1.0 nm within 10 ns. The representative snapshots of complexes at the end of simulations are shown as in Fig. 2a, and the complexes of estradiol derivatives (shown in green) and hERα LBD were compared with the original E_2_-hERα LBD crystal structure, where E_2_ is shown in magenta. The comparison confirmed that the estradiol derivatives maintained the binding conformation with hERα LBD in the MD simulation with the tail of the linking arm fully exposed. It also indicated that adding a flexible linker with appropriate length was an effective way not only to retain the capability being recognized by hERα LBD but also to avoid the possible steric hindrance caused by STV. The root-mean-square fluctuation (RMSF) is a measure of the atomic position deviation in a given length of time defined as the atomic position deviation during MD simulations^31^. The RMSF results further indicated that H12 (residues 531–549) was the most fluctuating part of hERα LBD, while H1–H11 and the ligand-binding pocket usually remained unaffected (Fig. S5). It could be another explanation for why the addition of linkers in the estradiol derivatives had a slight impact on the binding pocket of hERα LBD for estrogen identification.

**Table 1:** Performance comparison of this work with other reported nER-based biosensors for EE detection.

Transducer used	Quantitative range (ng ml^−1^)	LOD (ng ml^−1^)	Ref.
Electrochemical	/	/	^6^
Fluorescent	/	/	^13^
Fluorescent	20.8–476.7	1.05	^14^
Fluorescent	0.1–20	0.1	^48^
Piezoelectric	2.72–27.2	2.12	^18^
Nonisotopic	/	0.2	^10^
Surface plasmon resonance	2–6	0.2	^15^
Surface plasmon resonance	/	1	^49^
Surface plasmon resonance	/	1.4	^50^
Surface plasmon resonance	0.01–100	0.0015	This work

Note: “/” means undetectable or not available.

Binding energy in the simulations was calculated to predict the binding affinity of hERα LBD and estradiol derivatives. As shown in Fig. 2b, the complex of estradiol derivative 8 and hERα LBD exhibited the lowest average binding energy during the MD simulations, while the simulated binding energies of other derivatives–hERα LBD complex structures were much higher. The results of Hbond formation further confirmed that estradiol derivative 8 was able to form more hydrogen bonds to hERα LBD compared with other derivatives, including 11, 16, and 20 (Fig. 2c), therefore resulting in the higher binding affinity of the complex of estradiol derivative 8 and hERα LBD. Based on the above MD simulation results, estradiol derivative 8 with the most stable binding capability to hERα LBD was chosen for the synthesis of the E_2_-STV conjugate.

### Optimization of plasmonic sensing characteristics

**Fig. 3:**
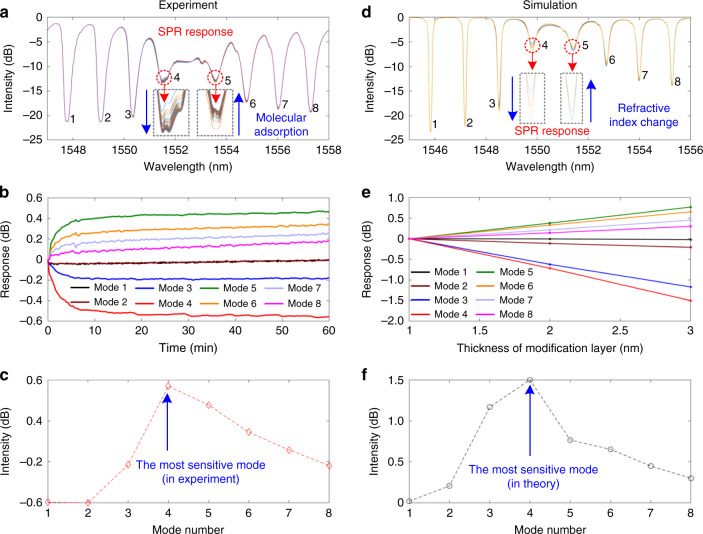
Optimization of plasmonic sensing characteristics. **a** Experimental SPR spectrum response of the plasmonic optical fiber when modified with the DTB-PEG-SH on the gold film in 1 h (inset: zoomed spectral changes of the two most sensitive modes 4 and 5); **b** Relative intensity change of the modes 1–8 during the whole DTB-PEG-SH modification; **c** Total intensity change of modes 1–8 after 1 h reaction; **d**–**f** The simulated results corresponding to the conditions in panels **a**–**c**, in good agreement with the experimental results.

To achieve the best sensing performance of plasmonic optical fiber, the most sensitive plasmonic mode located in the SPR envelope to monitor the interactions of molecules was investigated and validated both experimentally and in silico, the mechanism of which is shown in Fig. S6. Figure 3a shows the cladding mode change of plasmonic optical fiber when modified with the DTB-PEG-SH on the gold film in 1 h. As the SPR envelope has a red shift associated with increased RIs, i.e., more molecules attached, the amplitudes of the plasmonic cladding modes (marked as 1, 2, 3, 4) decreased while the other plasmonic modes (marked as 5, 6, 7, 8) increased, and all modes exhibited different relative intensity sensitivities. Among them, the two most sensitive modes 4 and 5 were identified and their zoomed spectral changes are shown in the inset. Figure 3b shows the relative intensity changes of the modes 1–8 during the whole reaction process. The mode 4 closely located at left shoulder of the SPR envelope exhibited the maximum relative intensity response for the DTB-PEG-SH adsorption. Figure 3c shows the total intensity changes of modes 1–8 after 1 h reaction, which exhibited a quantification curve similar to the profile of the SPR envelope.

For better comparison with the experimental data, the simulated results corresponding to the above experimental conditions were conducted. The simulation of the transmission spectrum of the fabricated gold film-coated TFBG was carried out by first solving for the modes of the fiber structure (inclusive of core, cladding, metal layers, high refractive index molecular, and water) with a complex vectorial finite-difference algorithm and then using the coupled mode theory as described previously^32^ for the transmission of TFBG with the aid of MATLAB. The parameters used in the simulation were as follows: a core radius of 4.1 μm with a RI of 1.4545, a cladding radius of 62.5 μm with a RI of 1.4467, a gold coating with a thickness of 50 nm and complex RI of 0.58−i×11, and a RI index of 1.3154 for water. The effective RI of surface molecular over gold coating is 1.48 (Fig. S7)^33^. Figure 3d shows simulated transmission spectra of the fabricated gold film-coated TFBG in DI water. It is worth mentioning that there was a sharp decrease in the amplitude of cladding mode resonances in the vicinity of 1550 nm, where those cladding modes had transferred energy to a lossy plasmon wave at the gold–water interface. It means that the gold–water interface is highly sensitive to the molecular interactions. The intensity changes of eight selected cladding modes responding to the thickness of the small molecular modification layer were demonstrated and exhibited the same trends with the experimental results, i.e., the decreased amplitudes of modes from 1 to 4 and the increased ones from 5 to 8. All modes showed different sensitivities and the most sensitive modes were also identified to be 4 and 5 and their zoomed spectral changes are shown in the inset. Figure 3e shows the responses of different SPR cladding modes to the thickness of small molecular modification layer from 1 to 3 nm. The mode 4 closely located at left shoulder of the SPR envelope was further confirmed with maximum relative intensity response, which accorded well with the above experimental results. The simulated relative intensities of each cladding modes to 3 nm modification layer (Fig. 3f) also exhibited the same pattern as the SPR envelope, as the experiment revealed.

To summarize, both the above experimental and simulated results identified that the highest sensitivity plasmonic mode of the fabricated gold film-coated TFBG to surface RI was mode 4, which was located at the left shoulder of the SPR envelope.

### Performance evaluation

As revealed above, the SPR envelope will shift in wavelength with the changed surface RI and then modulate the other phase-matched cladding modes. The wavelength shift of the SPR envelop was also observed under the different bulk solutions used in the analysis. As a consequence, the intensity response of the biosensor arose from both the biomolecule amount attached on the surface and the selected mode. The mode with the highest sensitivities was changing under different bulk solution conditions, hence possible to cover up the response caused by the target, especially at low concentrations. To mitigate the negative impact caused by the fluctuated bulk RI fluctuations of different samples, we calibrated and tested the reflected spectra under different E_2_ concentrations in the DI water. Under the same bulk solution conditions, the surface RI change caused by the EEs can be quantified with the intensity response of TFBG under the selected specific mode. More EEs competed with more E_2_–STV conjugates released from the resin-hERα LBD, which induced their more attachment and hence higher RIs on the TFBG surface. As a consequence, the stronger red shifted SPR envelope caused less attenuation of our selected resonance mode, hence resulting in the stronger relative intensity modulation of narrow cladding mode. All the above provides the theoretical basis for the ultrasensitive quantification of EEs.

**Fig. 4:**
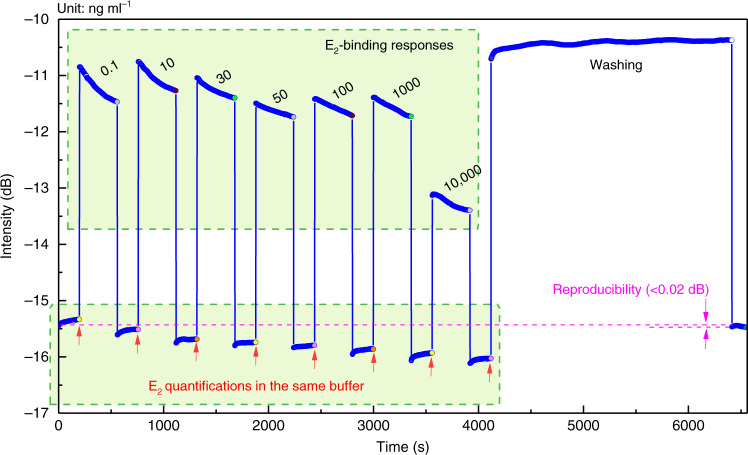
Stability of gold film-coated TFBG-based SPR biosensor using hERα LBD as a recognition element for EE detection. Representative real-time intensity curve responding to different concentrations of E_2_ and reproducibility by washing buffer.

Based on the optimized assay conditions (Fig. 3 and Figs. S8 and S9), the performance of TFBG-based SPR biosensor platform was evaluated by using E_2_ as the model analyte for EEs. Although 1% (w/v) sodium dodecyl sulfate (SDS) solution in phosphate-buffered saline (PBS) was reported to regenerate the antibody–antigen interaction on the nanocoated fiber surface^34^, the DTB-modified plasmonic optic fiber surface is expected to be stably regenerated by using the washing buffer (0.5% SDS, pH 1.9) in the light of the binding between STV and DTB^31^. We first tested the stability of temporal spectrum responses for different concentrations of E_2_ (Fig. 4). The representative real-time intensity curve revealed the stable and target-dependent optical response to E_2_ with the concentration varying from 0.1 ng ml^−1^ gradually increasing to 10,000 ng ml^−1^, with a dynamic range of more than five orders of magnitude. The signal was reproducible with attenuation <0.02 dB after the plasmonic optic fiber surface was regenerated.

**Fig. 5:**
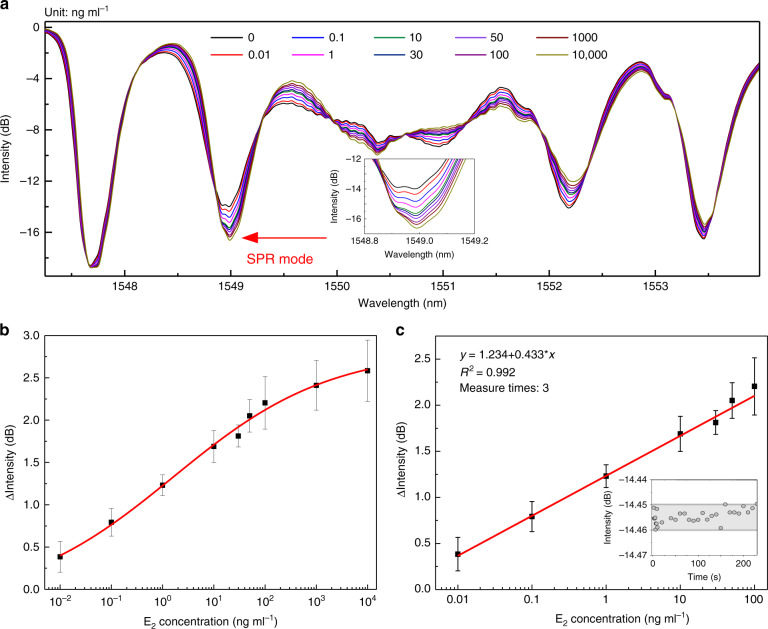
Sensitivities of gold film-coated TFBG-based SPR biosensor using hERα LBD as a recognition element for EEs detection. **a** Spectral response of SPR mode versus different E_2_ concentrations; **b** variations of ΔIntensity with the concentration of E_2_ and logarithmic calibration plot; **c** linear range and corresponding calibration plot to detect E_2_. Inset: intensity fluctuation of a blank sample. The error bars correspond to the standard deviation of the data points in triplicate experiments.

We further evaluated the feasibility of ultrasensitive detection for EEs via the TFBG-based SPR biosensor platform. The spectrum response of monitored SPR mode under different concentrations of E_2_ is recorded in Fig. 5a. It was observed that higher E_2_ concentrations were accompanied with lower optical intensities. To better quantify the targets, we use the optical intensity change (ΔIntensity, which is defined as the intensity difference between the base intensity in DI water before reaction and the intensity after certain reactions) to test the sensitivity of this biosensor for EEs.

We recorded the ΔIntensity of both spectra from 1545 to 1556 nm and mode 4 (inset of Fig. 5a) and related them to the concentrations of E_2_ (Fig. 5b). A logistic function-fitted calibration curve revealed the linear range of 0.01–100 ng ml^−1^ between the intensity and E_2_ concentration with a slope of 0.433 and linearity of 0.99 (Fig. 5c, inset: intensity fluctuation of the blank sample). A dramatically linear dynamic range of four orders of magnitude was achieved with a linear fitting function of *y* = 1.234 + 0.433*x. The limit of detection (LOD) of this biosensor toward E_2_, defined as the concentration in which the signal to noise ratio is 3, was calculated to be 1.5 × 10^−3^ ng ml^−1^, considering that the standard deviation of blank was 0.003 dB (inset of Fig. 5c)^35^. It was one order lower than that of the defined maximal E_2_ level in drinking water, i.e., 8.0 × 10^−2^ ng ml^−1^ set by the Japanese government. The LOD of this biosensor for EE detection was compared with other reported nER-based biosensors (Table 2), and the sensitivity superiority of this biosensor is impressive.

**Table 2:** Recovery of E_2_ in real-water samples using the TFBG-based SPR biosensor (*n* = 3).

Sample	Spiked E_2_ (ng ml^−1^)	Detected E_2_ (ng ml^−1^)	Recovery (%)	Coefficient variation (%)
Tap water	0	/	/	/
	1	0.91 ± 0.06	91	6
	10	11.23 ± 0.49	112	5
Pond water	0	/	/	/
	1	1.06 ± 0.10	106	10
	10	9.80 ± 0.69	98	7

Note: “/” means undetectable or not available.

We attributed the sensitivity superiority of this biosensor to the following reasons. Through compensating the red shift of the SPR envelope by using the same bulk solution for testing, the gold–water interface of this biosensor was proven to be highly sensitive to a small change of RIs. Moreover, the EE-induced sensing interface change was characterized by the biological macromolecule of E_2_-STV conjugate through the rational sensing design, which greatly amplified the signals.

The broad sensing capability of this biosensor arises from the specific binding capability between EEs and nERs. To demonstrate its capability for detecting broad ranges of EEs, we selected six phthalate esters, which were potential EEs widely reported by numerous studies^31,36^, for investigation. The normalized signal variations responding to 10,000 ng ml^−1^ phthalate esters, including butyl benzyl phthalate (BBP), diisopentyl phthalate (DIPP), diethyl phthalate (DEP), dimethyl phthalate (DMP), and dioctyl phthalate (DOP), compared with that caused by 10 ng ml^−1^ E_2_ are summarized in Fig. S10. The signal decreased in the order BBP > DIPP > DEP > DMP, indicating the relatively weaker estrogen-agonist potencies; however, DOP showed a comparable signal as the control sample, indicating no evidence of estrogenic activities measured by this hERα-based biosensor. The results obtained above are consistent with previous studies^31,36^.

Moreover, the interaction of hERα LBD with the investigated phthalate esters was simulated by molecular docking and MD. The complex of hERα LBD and four phthalate esters exhibited stable docking poses with the molecular docking scores (Dscores) in the order BBP < DIPP < DEP < DMP (Fig. S11a). The MD simulations also revealed that the binding energy of EE–hERα LBD complex structures showed the same changing trend as the Dscore (Fig. S11b). However, no stable docking pose or Dscore was generated for the complex of DOP and hERα LBD, indicating that DOP was not suitable for hERa LBD binding. The in silico studies that predicted the estrogen-agonist potencies of the phthalate esters are given as follows: BBP > DIPP > DEP > DMP, while the hERa-active potencies of DOP was negligible. The above in silico simulation results accorded well with the experimental results, which further confirmed the biosensing capability of this technique towards broad ranges of EEs.

To demonstrate its capability for practical and real-water samples, two types of real-water samples including laboratory tap water and pond water from the campus of Tsinghua University spiked with different concentrations of E_2_ were detected and evaluated by using the biosensing platform. Only the pond sample was filtered through a 0.45 μm filter (Millipore Corp., Bedford, MA) before spiking. Two spiked concentrations (1 ng ml^−1^, 10 ng ml^−1^) of E_2_ were chosen according to the sensitivity of our developed biosensor. The concentrations measured by this biosensing platform were compared with spiked concentrations, and results are listed in Table 3. When the non-spiked samples were pumped into the biosensor, no significant change in signal was captured, indicating that EEs were non-detectable in any samples by using this technique. For spiked samples, both for the case of low and high E_2_ concentrations, the calculated concentrations were close to original spiked values. Notably, the recovery rates in the pond water were more satisfactory than in the laboratory tap samples, considering that the tap water comes from deep groundwater sources and the hardness of this tap water is in the range of 290–350 μg ml^−1^ for calcium carbonate^37^. The high hardness could be one of the possible reasons affecting the interaction between hERα and EEs, hence the biosensor signal. In summary, the recovery rates of E_2_ ranged from 91 to 112%, demonstrating the satisfactory accuracy of this biosensor and indicating the application potential in real-water samples with a simple pretreatment.

**Table 3:** Eleven estradiol derivatives used in the simulation.

Number	Structure	Number	Structure
4		8	
11		16	
20		r3	
r5		r8	
r11		r16	
r20			

## Discussion

In summary, a label-free plasmonic gold film-coated TFBG-based SPR biosensor using hERα LBD as the biological recognition element was developed to achieve the ultrasensitive detection of EEs. An E_2_–STV conjugate consisting of an STV moiety and an E_2_ moiety was designed to compete with the EEs in the environmental samples to bind with the hERα LBD in the form of resin–hERα LBD complex. Once more EEs existed, more E_2_–STV conjugates were released. The STV moiety of the released conjugates bound with the DTB molecules modified on the TFBG, following with the stronger relative intensity modulation of narrow cladding mode. The “turn-on” sensing mode realized the detection of EEs down to 1.5 × 10^−3^ ng ml^−1^ EEQ with a linear range of four orders of magnitude.

The proposed in-fiber plasmonic biosensor shows a minimal cross-sensitivity to temperature and its fabrication does not impact the structural integrity of the fiber. The biosensor is able to perform for the in-field continuous detection of estrogenic endocrine disruptors, meeting the highly desired demand for the timely monitoring of environmental status. Meanwhile, integrating such a fiber sensor with a hypodermic needle on the other hand would allow similar measurements, as portable on-site and in-field analysis in health monitoring. To realize a portable instrumentation for in-field measurement, we can use a real-time interrogation scheme by replacing the wavelength interrogation by optical power detection. In this case, a tunable laser (TLS) can be used as a source instead of broadband light source (BBS), together with a photodiode as detector and an analog-to-digital converter to obtain the desired data (to replace the optical spectrum analyzer (OSA)). The function of the TLS is matching the wavelength of the most sensitive SPR mode of the TFBG, so once the sensor is characterized it can be replaced by a common laser (for example, a compact VCSEL). This technique relies on the principle of edge filtering so that the optical power change is produced as a result of the wavelength shift of the mode with respect to the fixed wavelength of the laser source.

Moreover, the platform demonstrated here was able to be extended for detecting a variety of endocrine disruptors by using different nuclear receptors. Endocrine disruptors are chemical compounds that mimics or interferes with the normal actions of hormones in the body via the specific interaction with the nuclear receptors. Bearing the similar biochemical characteristics with ER, other nuclear receptors, such as androgen receptor, thyroid receptor, and progesterone receptor, are expected to be integrated with the TFBG-based SPR biosensing platform for ultrasensitive detection of other endocrine disruptors.

Last but not least, true multidisciplinary efforts between photonics and bio- and electro-chemistry groups have led to impressive environmentally and clinically^38^, together with emerging renewable energy^39–42^, relevant applications for many substances that require detection and quantification. It is these efforts that are allowing the full potential of such optical fiber sensors to be reached and be able to exploit the extraordinary “Resolution + Specificity + Reproducibility” with fiber gratings and SPR in simple configurations and the combination of other advanced sensing technologies^43–45^.

## Materials and methods

### Materials and reagents

DTB-PEG-SH-5K was bought from NANOCS. Ni–NTA agarose resin was purchased from GE. SDS, E_2_, isopropyl-β-d-thiogalactopyranoside, dimethyl sulfoxide, β-mercaptoethanol, nonidet P400, BSA, SDS, and other chemicals used for buffers and solvents were purchased from Sigma-Aldrich. All buffers were prepared by DI water (18.2 MΩ cm).

### Gold film-coated TFBG-based SPR biosensor platform

**Fig. 6:**
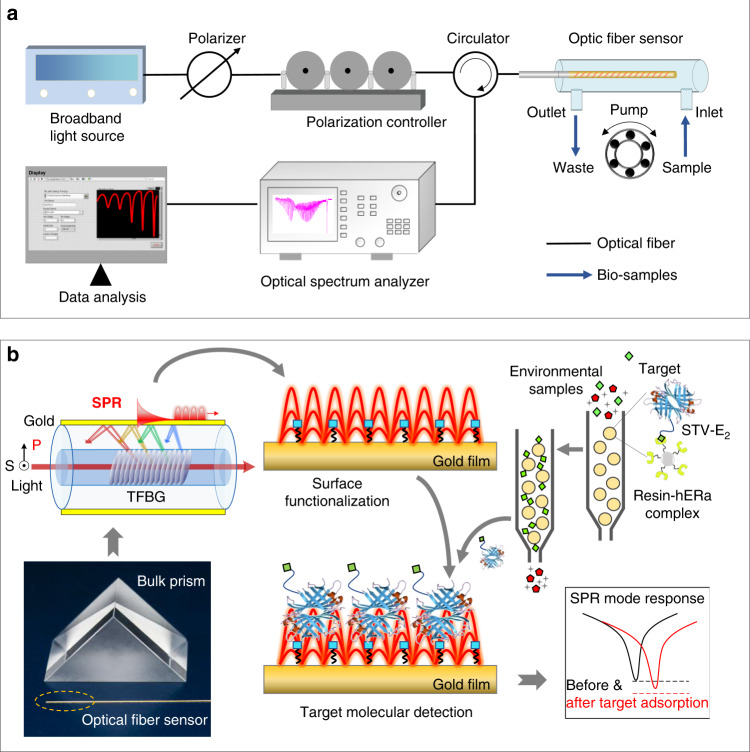
Plasmonic gold film-coated TFBG-based SPR biosensor using hERα LBD for ultrasensitive EE detection. **a** Schematic diagram of the biosensor set-up; **b** schematic illustration of the sensing mechanism for detection of broad ranges of EEs by using hERα LBD as the biological recognition element. In brief, the gold film-coated fiber surface is tethered with the DTB molecules, and the unbound E_2_–STV conjugates are captured via the STV–DTB affinity interaction into the range of SPR in red curve, which will change the spectral of fiber sensors.

Figure 6a shows the experimental set-up of gold film-coated TFBG-based SPR biosensor. The plasmonic TFBG was excited by a BBS (Golight, OS-EB-S-D-1450-400-30-0-FA) with the wavelength range of 1250–1650 nm (power density of −10 dBm nm^−1^) to provide superfine plasmonic spectral combs. Its reflection spectrum was monitored by an OSA with a high wavelength resolution of 0.02 nm (Yokogawa, AQ6370B). The signals were recorded continuously every 20 s using data analyzer, which was coded in our laboratory using LabVIEW. A linear polarizer and a polarization controller were placed upstream of the circulator to adjust and orient the state of polarization of light launched into the fiber grating so as to provide the strongest SPR excitation. The photograph of self-developed SPR biosensor is shown in Fig. S12.

To fabricate the gold film-coated TFBG, 1-cm-long TFBG was inscribed in the core of boron germanium co-doped highly photosensitive single-mode fiber (FIBERCORE PS1250/1500) using a ~1110 nm period uniform phase mask and a laser emitting at 193 nm (Bragg Star Industrial, Coherent, Inc.). TFBG with core mode at 1615 nm and tens of cladding modes from 1500 to 1600 nm were achieved. Then an ultra-thin gold film was deposited on the TFBG probe using the sputtering technique (Sky Technology Development, China). To achieve a high-quality coating on the TFBG surface, a layer of chromium with 2–3 nm thickness was used as a binder in between the silica fiber and the gold film. To ensure a uniform thickness of gold film over the whole fiber surface, the fiber was rotated along its axis during the sputtering. With such design, a robust and uniform gold film with a thickness of 50 nm was coated over the fiber surface. The gold film thickness was further confirmed by using an ellipsometer (Optical Thickness Meter, Otsuka Electronics). The tilt of the grating is an important parameter that can be used to choose which set of cladding modes is going to be excited. As a result, it makes it possible to adjust the operating range of the sensor in order to optimize the response for certain refractive indices. Here the gratings had a tilt of 12°, which maximizes the amplitude of the resonances in aqueous solutions with refractive indices around 1.32–1.34. Finally, the sensor is specially designed with a gold mirror at the end of the fiber. This simple additional process not only offers an increased broadband reflectivity to near 100% but also makes the light going through the grating twice and doubling the attenuation of each resonance. This configuration also ensures strain-free operation of the sensor to eliminate the effect of cross-sensitivity to strain of the higher-order cladding and plasmonic modes when the sensor is closely attached.

### Sensing mechanism

Figure 6b depicts the schematic illustration of the TFBG-based SPR biosensor for the detection of broad ranges of EEs. It is worth mentioning that, as a classic subtype of nERs, nERα protein shows estrogen-dependent gene transcriptional activation via their key activation domain–LBD^1,5,7^. Structurally, nERα LBD is very conserved and is responsible for the ligand binding via identification of the structural commonalities among diverse estrogenic endocrine disruptors^5,46^. To realize the recognition of broad ranges of EEs, the LBD of human nERα (hERα LBD) (aa302–552 with three mutations of aa381, 417, 530 from Cys to Ser) encoding plasmid (pET 28a) was self-constructed in our laboratory and expressed in *Escherichia coli* (BL21 DE3). By adding six His tag to its N-terminal, it was purified by the affinity chromatography via binding to the Ni^2+^-charged agarose (Ni–NTA His·Bind® resin) with an average diameter of 90 μm. The purified 31 kDa hERα LBD protein was confirmed by SDS-polyacrylamide gel electrophoresis as shown in Fig. S13. More details are described in our previous studies^14^. The obtained complex of resin–hERα LBD was used as the biological recognition element to avoid the biological activity loss of the eluted hERα LBD protein. An E_2_–STV conjugate consisting of a STV protein moiety and an E_2_ small-molecule moiety was designed to compete with the EEs in the environmental samples to bind with the hERα LBD in the form of bound state (resin–hERα LBD complex). Once more EEs existed, more E_2_–STV conjugates were released. After a simple centrifugal separation, the supernatant with unbound conjugates was fed into the flow cell of the TFBG-based SPR biosensor. The STV moiety of conjugates bound with the DTB molecules modified on the optical fiber via the STV–DTB affinity interaction^47^, which was followed with an increase in the intensity of cladding mode with SPR excitation. Normally, the more the EEs, the more red shift the SPR envelope, resulting in the stronger relative intensity modulation of narrow cladding mode. It demonstrated a typical “turn-on” sensing mode.

### Design of E_2_–STV conjugate

To realize the conjugate-mediated measurement for the quantification of EEs in environmental samples, the E_2_–STV conjugate that was able to interact with the hERα LBD efficiently was rationally designed. In order to avoid the steric hindrance caused by STV, various link arms were introduced to the E_2_–STV conjugates, containing five estradiol derivatives with flexible joints named 4, 8, 11, 16, and 20 and six estradiol derivatives with rigid joints named r3, r5, r8, r11, r16, and r20 as shown in Table 3. The interaction of estradiol derivatives and hERα LBD were simulated by MD simulation, respectively. The crystal structure of E_2_-hERα LBD (PDB ID: 1GWR) obtained from the protein database RCSB PDB (http://www.rcsb.org) was first checked in Autodock Tools (http://autodock.scripps.edu/resources/adt) to remove unnecessary water molecules and add lost hydrogen atoms. Next, 11 kinds of estradiol derivatives were aligned to the original E_2_-hERα LBD crystal structure. Then the complexes of estradiol derivatives and hERα LBD were used as the initial conformation for MD simulation, which was carried out in Gromacs 2016.4 under the GROMOS96 43a1 force field. In the MD simulations, the complex was first solvated with simple point charge water molecules in a dodecahedral box and the minimum distance between complexes and the solvent box was 1.5 nm. The Na^+^ and Cl^−^ ions were then added to the solvent box for charge balance. All MD processes underwent energy minimization, temperature coupling (300 k), pressure coupling (1 bar), and 10 ns simulation. The estradiol derivative with the most stable binding capability to hERα LBD was chosen for the synthesis of the conjugate by Shanghai Qianyan Biotechnology Co., Ltd. (China). The nuclear magnetic resonance identification report of the synthesized estradiol derivative 8 is demonstrated in Fig. S14.

### Surface functionalization of plasmonic optical fiber sensor

To realize the signal record of binding the conjugate on the surface of TFBG, the plasmonic optical fiber with gold film on the surface was further modified with the DTB molecule, which showed specific binding with the STV moiety of E_2_–STV conjugate. Notably, the moderate binding affinity between STV and DTB enabled the regeneration of the fiber surface for reuse with less loss of activity, thereby allowing semi-continuous automated water monitoring. We used a well-established strategy to immobilize functional groups/molecules onto thin SPR films by synthesizing thiol-end DTB derivative (DTB-PEG-SH), forming self-assembled monolayers on gold film surfaces via Au-S bond (Fig. 1a). Briefly speaking, the gold-coated fiber surface was first treated by oxygen plasma for 5 min to remove possible contaminants and then thoroughly rinsed with DI water. The clean fiber was subsequently treated with 2 ml DTB solution (40 μM DTB-PEG-SH and 200 μM H_3_PO_4_) at a flow rate of approximately 1 ml min^−1^ for 3 h at room temperature to form the binding layer and then thoroughly rinsed with 10 mM PBS, pH 7.4. Finally, it was immersed in the BSA solution (5 mg ml^−1^ in 10 mM PBS buffer, pH 7.4) for 1 h to block its non-specific binding sites. The surface-functionalized gold-coated TFBG fiber was stored at 4 °C before use.

### Quantification of EEs

After the optimization of procedures for EEs detection, 500 μl of 5 μg ml^−1^ E_2_–STV conjugate was first added into 58 pmol resin–hERα LBD, and the mixture was maintained at 4 °C overnight for the affinity binding of E_2_ with hERα LBD. Next, the mixture was centrifuged at 3000 rpm for 2 min and 450 ml of supernatant was discarded. Subsequently, 500 μl of E_2_ standard solutions or test samples were added and rotationally mixed at 4 °C for 8 h to compete for the E_2_-binding motif with hERα LBD. The dissociated conjugate was then introduced to bind the DTB on the TFBG fiber surface for 6 min, which was detected by the SPR excitation after stabilizing the probe through the DI water. After the signal detection, the E_2_–STV conjugate was able to be eluted using 5 ml of 0.5% SDS (pH 1.9) for 25 min to ensure the regeneration of sensing surface. The whole detection procedure took place within 40 min.

The amount of EEs in test samples was represented by the estrogenic activities, which was characterized by the EEQ by using E_2_ as the reference compound.

## Supplementary information

Revised Supplementary Information

## Data Availability

All data needed to evaluate the conclusions in the paper are present in the paper and/or the Supplementary Materials. Data from these experiments and MATLAB code used for data analyses are available from the corresponding authors upon reasonable request.
