# Supplementary material for: Ultrasensitive detection of endocrine disruptors via superfine plasmonic spectral combs
Source: Light Sci Appl. 2021 Sep 7;10:181. doi: 10.1038/s41377-021-00618-2 (PMC8423748; doi:10.1038/s41377-021-00618-2)
Supplement: Supplementary file 1 — Revised Supplementary Information [file 41377_2021_618_MOESM1_ESM.docx]

**Supplementary Information for**

**Ultrasensitive Detection of Endocrine Disruptors via Superfine Plasmonic Spectral Combs**

**Authors**

Lanhua Liu^1#^, Xuejun Zhang^2#^, Qian Zhu^1#^, Kaiwei Li^2^, Yun Lu^1^, Xiaohong Zhou^1*^ and Tuan Guo^2*^

**Affiliations**

^1^ *State Key Joint Laboratory of ESPC, School of Environment, Tsinghua University, Beijing 100084, China*

^2^ *Institute of Photonics Technology, Jinan University, Guangzhou 510632, China*

^*^Correspondence and requests for materials should be addressed to Xiaohong Zhou ([xhzhou@mail.tsinghua.edu.cn](mailto:xhzhou@mail.tsinghua.edu.cn)) or Tuan Guo ([tuanguo@jnu.edu.cn](mailto:tuanguo@jnu.edu.cn)).

^#^These authors contributed equally to this work.

**Caption of Figures**

**Figure S1** A glance at Estrogen detection technology as reported from references.

**Figure S2** AFM topography images of (a) bare and (b) DTB-PEG-SH modified fiber, including a 500 nm × 500 nm plane AFM image (Higher left), cross-section height variations taken at a rough area (Lower: position indicated by white grid line in the topographic images).

**Figure S3** Molecular docking of six rigid estradiol derivatives with hERα LBD: r3 (yellow), r5 (tints), r8 (slate), r11 (megentas), r16 (cyan), and r20 (grey).

**Figure S4** Molecular docking of estradiol derivative 4 with hERα LBD.

**Figure S5** RMSF of the main chain atoms of estradiol derivatives occupied hERα LBD.

**Figure S6** Illustration of how the shift of the SPR envelope (dash curves) introduces amplitude changes in the neighboring TFBG resonances in the transmission spectra (solid curves).

**Figure S7** Theoretical and experimental surface functionalization configuration: (a) a mixture of molecular and the buffer solution in the experiment; (b) a uniform film (with close RI to that of experimental case) over fiber surface in the simulation.

**Figure S8** Optimizations of E_2_/STV ratio (signal recovery efficiency equals *I/I_0_*, where *I_0_* and *I* represent the initial optical intensity and optical intensity after adding E_2_, respectively).

**Figure S9** Relationship between the intensity difference and the E_2_–STV conjugate concentration.

**Figure S10** Normalized signal variations responding to different potential EEs. 10000 ng ml^-1^ phthalate esters, including Butyl benzyl phthalate (BBP), Diisopentyl phthalate (DIPP), Diethyl phthalate (DEP), Dimethyl phthalate (DMP), and Dioctyl Phthalate (DOP). 10 ng ml^-1^ E_2_ was used as a reference.

**Figure S11** Molecular docking and MD simulations between potential EEs and hERa. (a) Docking scores (Dscores) and (b) binding energy of the complex of potential EEs with hERα LBD, using E_2_ as a reference ligand.

**Figure S12** Instrument photograph of TFBG-based SPR biosensor platform.

**Figure S13** Characterization of hERα LBD protein by SDS-PAGE. Lane M: molecular weight standards (marker in kDa); Lane 1: cell lysis solution of induced E. coli hERα LBD; Lane 2: effluent after purification by Ni-NTA affinity column; Lane 3: purified hERα LBD.

**Figure S14** Structures of the estradiol derivatives assigned by NMR provided by the company.

**S1. Estrogen detection technology**

**Figure S1** A glance at Estrogen detection technology as reported from references^1-8^.

This figure pictures the diversity of technologies applied in the estrogen detection field. The various techniques are given with the corresponding size of the pie quantifying the percentage of the relevant published articles found in the bibliography for each of the techniques.

**References**

1. Rodriguez-Mozaz, S. et al. Biosensors for environmental monitoring of endocrine disruptors: a review article. *Analytical and Bioanalytical Chemistry* **378**, 588-598 (2004).
2. Habauzit, D. et al. Determination of estrogen presence in water by SPR using estrogen receptor dimerization. *Analytical and Bioanalytical Chemistry* **390**, 873-883 (2008).
3. Liu, Y. et al. Detection and identification of estrogen based on surface-enhanced resonance Raman scattering (SERRS). *Molecules* **23**, 1330-1340 (2018).
4. Scognamiglio, V. et al. Analytical tools monitoring endocrine disrupting chemicals. *TrAC - Trends in Analytical Chemistry* **80**, 555-567 (2016).
5. Cevenini, L. et al. A novel bioluminescent NanoLuc yeast-estrogen screen biosensor (nanoYES) with a compact wireless camera for effect-based detection of endocrine-disrupting chemicals. *Analytical and Bioanalytical Chemistry* **410**, 1237-1246 (2018).
6. Hennies, M. et al. Vitellogenin in carp (Cyprinus carpio) and perch (Perca fluviatilis): purification, characterization and development of an ELISA for the detection of estrogenic effects. *Science of the Total Environment* **309**, 93-103 (2003).
7. Arnold, S. F. et al. A yeast estrogen screen for examining the relative exposure of cells to natural and xenoestrogens. *Environmental Health Perspectives* **104**, 544-548 (1996).
8. Liu, L. H. et al. Facile screening of potential xenoestrogens by an estrogen receptor-based reusable optical biosensor. *Biosensors and Bioelectronics* **97**, 16-20 (2017).

**S2. AFM topography cross sections of the bare and chemically modified fiber surfaces**

**Figure S2** AFM topography images of (a) bare and (b) DTB-PEG-SH modified fiber, including a 500 nm × 500 nm plane AFM images (left), cross-section height variations taken at a rough area (right: position indicated by white grid line in the topographic images)

**S3. Molecular docking of six rigid estradiol derivatives with ER-LBD**


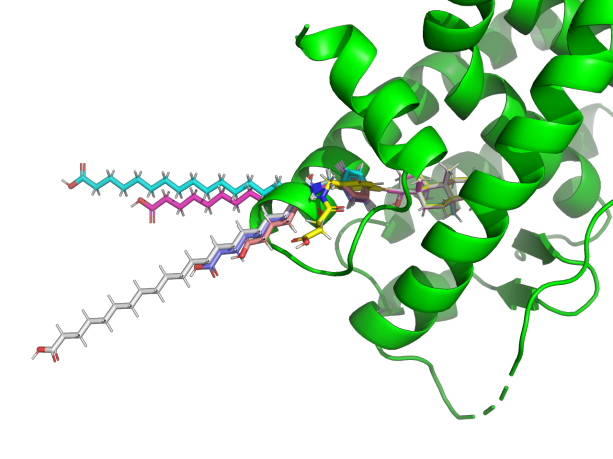


**Figure** **S3** Molecular docking of six rigid estradiol derivatives with hERα LBD: r3 (yellow), r5 (tints), r8 (slate), r11 (megentas), r16 (cyan) and r20 (grey)

**S4. Molecular docking of estradiol derivative 4 with ER-LBD**


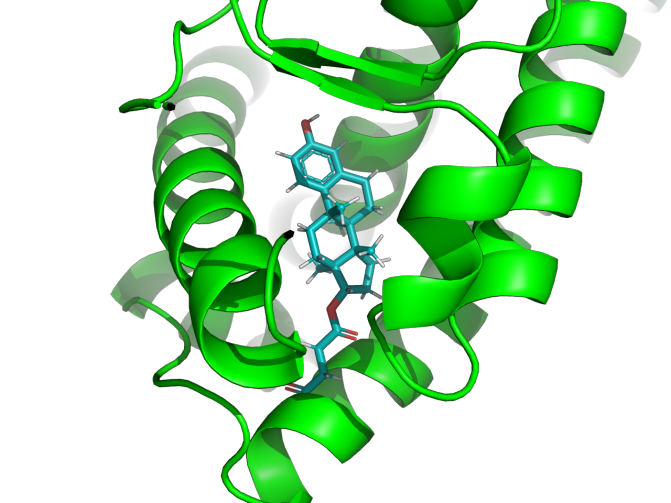


**Figure S4** Molecular docking of estradiol derivative 4 with hERα LBD

**S5. Molecular dynamics simulations**

**Figure S5** RMSF of the main chain atoms of estradiol derivatives occupied hERα LBD (estradiol derivatives 8, 11, 16, and 20 with different flexible carbon skeleton was shown in Table 1)

**S6. Sensing mechanism of plasmonic TFBG**

**Figure S6** Illustration of how the shift of the SPR envelope (dash curves) introduces amplitude changes in the neighboring TFBG resonances in the transmission spectra (solid curves)

**S7. Experimental and simulated model for plasmonic sensing characterization**

**Figure S7** Theoretical and experimental surface functionalization configuration: (a) a mixture of molecular and the buffer solution in the experiment; (b) a uniform film (with close RI to that of experimental case) over fiber surface in the simulation.

**S8. Optimization of E_2_/STV ratio**

To achieve an optimal detection performance, the E_2_/STV ratio was optimized by selecting the mole concentration ratios for synthesising reaction, that is, n(c(E_2_):c(STV)), to be 10, 20, 50 and 100, the corresponding E_2_/STV conjugate coupling ratios can be measured using the matrix-assisted laser desorption/ionisation–time-of-flight (MALDI–TOF) mass spectrometry, as shown by the black line in Figure S8.

Approximately 0.5 mL of 0.5 μg ml^-1^ Cy5.5-labelled conjugates with different coupling ratios are mixed with equimolar resin–hERα at 4 °C overnight, the supernatant is discarded, and equimolar E_2_ standard solution is subsequently added to compete with the resin–hERα. The competing and initially added conjugates are separately introduced into the sensing surface, and the signals are recorded (*I* and *I_0_*). The signal recovery efficiencies, that is, *I*/*I_0_*, reach a maximum at n(c(E_2_):c(STV)) of 20, which is selected as the optimised value for synthesis.

**Figure S8** Optimizations of E_2_/STV ratio (signal recovery efficiency equals *I/I_0_*, where *I_0_* and *I* represent the initial optical intensity and optical intensity after adding E_2_, respectively).

**S9. Optimization of conjugate concentration**

For the conjugate concentration optimization, to ensure that the resin–hERα LBD fully combined with the E_2_–STV conjugate, we tested the intensity of the E_2_–STV conjugate at different incubating concentrations with 0 pmol resin–hERα and 58 pmol resin–hERα. Fig. S12 shows the difference of optical intensity (ΔI) under both conditions, which is used for the optimization of the E_2_–STV conjugate concentration. The intensity difference increases to a maximum and slightly changes when the E_2_–STV conjugate concentration reaches 5 μg ml^-1^, indicating that the resin–hERα LBD binding sites are fully saturated. Thus, 5 μg ml^-1^ conjugate is used to ensure the highest sensitivity.

**Figure S9** Relationship between the spectral intensity difference and the E_2_–STV conjugate concentration.

**S10.** **Normalized signal variations responding to different potential EEs**

**Figure S10** Normalized signal variations responding to different potential EEs. 10000 ng ml^-1^ phthalate esters, including Butyl benzyl phthalate (BBP), Diisopentyl phthalate (DIPP), Diethyl phthalate (DEP), Dimethyl phthalate (DMP), and Dioctyl Phthalate (DOP). 10 ng ml^-1^ E_2_ was used as a reference.

**S11. Molecular docking and MD simulations between potential EEs and hERa**

**Figure S11** Molecular docking and MD simulations between potential EEs and hERa. (a) Docking scores (Dscores) and (b) binding energy of the complex of potential EEs with hERα LBD, using E_2_ as a reference ligand.

**S12. The photograph of the self-developed SPR biosensor**

**Figure S12** Instrument photograph of TFBG-based SPR biosensor platform

**S13. Characterization of hERα protein by SDS-PAGE**

**Figure S13** Characterization of hERα LBD protein by SDS-PAGE. Lane M: molecular weight standards (marker in kDa); Lane 1: cell lysis solution of induced E. coli hERα LBD; Lane 2: effluent after purification by Ni-NTA affinity column; Lane 3: purified hERα LBD

**S14. Nuclear magnetic resonance (NMR) identification report of the synthesized estradiol derivatives with flexible joints named 8**


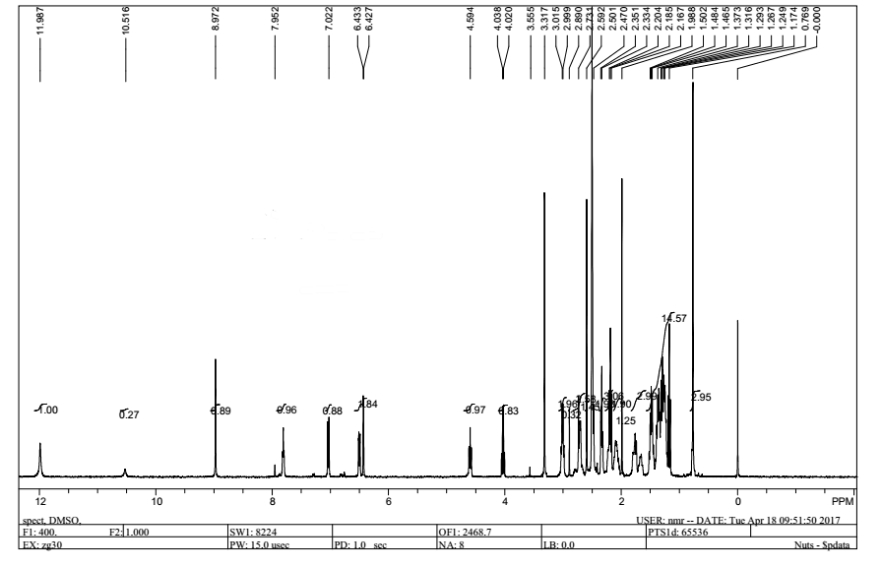


**Figure S14** Structures of the estradiol derivatives assigned by NMR provided by the company
